# Memoriam for Renaud Mahieux

**DOI:** 10.1186/s12977-021-00551-7

**Published:** 2021-03-17

**Authors:** Fatah Kashanchi, Ali Bazarbachi, Antoine Gessain

**Affiliations:** 1 George Mason University, Manassas, VA 20110 USA; 2 American University of Beirut, Beirut, Lebanon; 3 Institut Pasteur, Paris, France


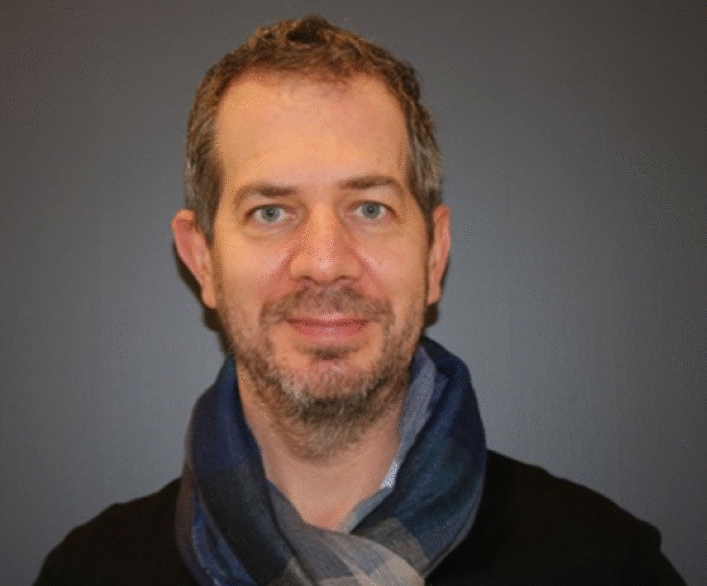


Renaud Mahieux passed away on Wednesday, December 9 2020, following a relapse of a long illness and a new infection with Covid-19. He was 52 years old and was survived by his wife Mathilde and his two children Guillemette and Barthelemy.

Renaud’s scientific career began in 1991, at the Pasteur Institute in Paris, where he completed his “Diplome d’Etudes Approfondies” (DEA) internship in Virology. It was then that he met Antoine Gessain, who had just returned from a post-doctoral internship at the National Institute of Health (NIH) and who welcomed him in the laboratory of Pr. Guy de Thé’s unit. This marked the beginning of a strong scientific journey culminating in great discoveries in the retrovirology field, and a solid friendship that never wavered. This opportunity was the first exposure of Renaud to the HTLV-1 research and traced the beginning of his scientific path.

Renaud obtained his Ph.D in 1997 in Microbiology—Molecular Virology at the University of Paris 6, under the supervision of Pr. Guy De Thé and Pr. Antoine Gessain. He focused on the genetic diversity of HTLV-1 and its related STLV-1 simian viruses. At that time, Renaud also participated with Antoine Gessain and Philippe Mauclère, a Virologist at the Centre Pasteur of Cameroon, in one of the first missions of the unit, in the search of retroviral variants in village populations living in remote forest areas of southern Cameroon. During his stay in Guy de Thé's unit, Renaud met Dr. Mirdad Kazanji, who is currently Director of the Pasteur Institute in French Guyana, and became a close friend and colleague for many years. Subsequently, Renaud pursued his post-doctoral fellowship at NIH, in the laboratory of Dr. John Brady (NIH/NCI) and focused on the molecular biology of HTLV-1. His collaboration with Dr. Brady resulted in more than 20 research papers that addressed several molecular topics in HTLV-1 pathophysiology, encompassing the viral onconprotein Tax, the tumor suppressor protein p53, the NF-κB signaling pathway regulation and apoptosis in HTLV-1 infected cells. During his stay at the NIH, he worked closely with other colleagues in the field of HTLV-1, namely Drs. Steve Jacobson and Fatah Kashanchi, who became some of his lifelong and close colleagues and friends. After his postdoctoral position at the NIH, he returned to France where he fulfilled a position at the Institut National de la Santé et de la Recherche Médicale (INSERM), as a Researcher in the Epidemiology and Physiopathology of Oncogenic Virus unit at the Pasteur Institute. Thereafter, Renaud continued his fruitful career with Pr. Gessain, after the retirement of Pr. Guy de Thé, and was instrumental in multiple exciting discoveries including the retrovirus HTLV-3, its zoonotic origin, and its molecular characteristics, the new simian viruses of the STLV-1 and STLV-3 types, and the understanding of the indeterminate HTLV serologies, which are very frequent in tropical areas. His brilliant scientific path was further marked by more than sixty publications, with his mentor and an NIH grant with Dr. Kashanchi on HTLV-3. It was also at this time, when he began to take part in teaching at the Pasteur Institute. Virology was indeed a topic he liked very much and he fully developed in Lyon.

In 2008, Renaud joined the École Normale Supérieure (ENS) of Lyon where he fulfilled a Professorial position. This marked the beginning of the second arm of his career where his passion to research and teaching were highlighted by the pursuit of quality research work in the field of HTLVs, and the development of a passionate teaching path in the field of Virology. In 2013, he became the head of the Biology Department of the ENS, after many accomplishments in a short period of time in ENS, reflecting his didactic and outstanding capacities. He indeed brought together a strong and dedicated teaching team, he developed scientific exchange programs with Japan and in particular with the team of Pr. Toshi Watanabe, he worked tirelessly with colleagues including Hélène Dutarte, and mentored talented students including Chloé Journo, who were inspired by his successful career, and are currently pursuing a similar brilliant path in the field of Retrovirology. Renaud earned an international recognition and became a world authority in retrovirology. He actively participated in the International Retrovirology Association (IRVA), of which he was secretary for many years. A testimony of his international networking was his relationship with Lebanon and a lifelong friendship with Pr. Ali Bazarbachi. Renaud visited Lebanon on numerous occasions where he presented research seminars, initiated research collaborations, was heavily involved in virology teaching, and served as member of Ph.D thesis committees.

Renaud was a world-class retrovirologist and co-authored more than 140 publications. He was invited as keynote speaker and chaired or co-chaired a large number of sessions at international meetings. He served on the editorial board and as reviewer for prestigious journals in the field of virology. Renaud had a warm and winning personality that fostered independence. People meeting him for the first time were often surprised to see how discreet and unpretentious he was. He spoke and wrote with great eloquence and precision, reflecting his hard work and clear thinking as a leader.

Renaud taught us that honesty, hard work, determination and optimism are the ingredients for great success in science and life. He was a beautiful soul, loyal in friendship, animated and expressing himself by a constant positive spirit. In discussing Renaud’s untimely death with Dr. Steve Jacobson of the NIH and many others in the field of HTLV-1 in recent past history, including Drs. Dale McFarlin, Pamela Rodgers-Johnson, Ralph Grassmann, John Brady, David Derse, Bill Harrington, Kuan-Teh (Teh) Jeang, Guy de Thé, Kazunari Yamaguchi, and Jean-Claude Vernant, he reminded us of the following inspiring passage:

“We are like dwarfs sitting on the shoulders of giants. We see more, and things that are more distant, than they did, not because our sight is superior or because we are taller than they, but because they raise us up, and by their great stature add to ours.”

John of Salisbury, 1159

Fatah Kashanchi.

Ali Bazarbachi.

Antoine Gessain.

